# In vitro activity and mode of action of phenolic compounds on *Leishmania donovani*

**DOI:** 10.1371/journal.pntd.0007206

**Published:** 2019-02-25

**Authors:** Christine Achiaa Antwi, Cynthia Mmalebna Amisigo, Jonathan Partt Adjimani, Theresa Manful Gwira

**Affiliations:** 1 West African Centre for Cell Biology of Infectious Pathogens, College of Basic and Applied Sciences, University of Ghana, Legon, Accra, Ghana; 2 Department of Biochemistry, Cell and Molecular Biology, College of Basic and Applied Sciences, University of Ghana, Legon, Accra, Ghana; Pasteur Institute of Iran, ISLAMIC REPUBLIC OF IRAN

## Abstract

**Background:**

Leishmaniasis is a disease caused by the protozoan parasite, *Leishmania*. The disease remains a global threat to public health requiring effective chemotherapy for control and treatment. In this study, the effect of some selected phenolic compounds on *Leishmania donovani* was investigated. The compounds were screened for their anti-leishmanial activities against promastigote and intracellular amastigote forms of *Leishmania donovani*.

**Methodology/Principal findings:**

The dose dependent effect and cytotoxicity of the compounds were determined by the MTT assay. Flow cytometry was used to determine the effect of the compounds on the cell cycle. Parasite morphological analysis was done by microscopy and growth kinetic studies were conducted by culturing cells and counting at 24 hours intervals over 120 hours. The cellular levels of iron in promastigotes treated with compounds was determined by atomic absorption spectroscopy and the effect of compounds on the expression of iron dependent enzymes was investigated using RT-qPCR.

The IC_50_ of the compounds ranged from 16.34 μM to 198 μM compared to amphotericin B and deferoxamine controls. Rosmarinic acid and apigenin were the most effective against the promastigote and the intracellular amastigote forms. Selectivity indexes (SI) of rosmarinic acid and apigenin were 15.03 and 10.45 respectively for promastigotes while the SI of 12.70 and 5.21 respectively was obtained for intracellular amastigotes. Morphologically, 70% of rosmarinic acid treated promastigotes showed rounded morphology similar to the deferoxamine control. About 30% of cells treated with apigenin showed distorted cell membrane. Rosmarinic acid and apigenin induced cell arrest in the G0/G1 phase in promastigotes. Elevated intracellular iron levels were observed in promastigotes when parasites were treated with rosmarinic acid and this correlated with the level of expression of iron dependent genes.

**Conclusions/Significance:**

The data suggests that rosmarinic acid exerts its anti-leishmanial effect via iron chelation resulting in variable morphological changes and cell cycle arrest.

## Author summary

The available drugs used in treatment of leishmaniasis, a disease caused by the *Leishmania* parasite, include pentavalent antimonials and amphotericin B. The cost and associated toxicity of the drugs, side effects and the emergence of drug resistance parasites, as well as the discomfort in drug administration suggests the need for new and better chemotherapeutic interventions. Iron is involved in several biological processes in the *Leishmania* parasite and it’s important for its growth and survival. The effects of using iron chelators to deprive parasite of iron was investigated in this study. Treatment of *L*. *donovani* with rosmarinic acid and apigenin inhibited the growth of promastigotes and intracellular amastigotes in a dose dependent manner. Morphologically, the compounds induced alterations in the parasites upon treatments. Rosmarinic acid was observed to cause the dysfunction of the mitochondria and alter the expression of iron dependent enzymes. Rosmarinic acid ability to chelate iron could be responsible for the changes in cell morphology and cell cycle observed.

## Introduction

Leishmaniasis is caused by the parasitic, single-cell eukaryotic organism called *Leishmania*. It is transmitted to man and animals (e.g. rodents, hydrax, canids) via a blood meal feed of the female sand-fly [1]. Currently, there are about 18 different *Leishmania* species including *Leishmania donovani* that have been discovered to be pathogenic to humans [2, 3].

*L*. *donovani* amongst other species of the parasite causes visceral leishmaniasis (VL). VL is the most intense and fatal clinical manifestation of the disease compared to the other form of leishmaniasis known as cutaneous leishmaniasis. The reported global annual mortality caused by VL infection is about 20,000 [3, 4]. It is the next cause of parasite-related death after malaria [1] and is thought to be underreported mainly due to subclinical forms, socioeconomic constraints and other barriers such as diagnosis and detection of the parasite. The disease remains a global threat that requires effective chemotherapy since not much progress has been made in the development of a potent vaccine. The available drugs used in the treatment of leishmaniasis include first line treatment drugs such as pentavalent antimonials and second line drugs (amphotericin B, pentamidine, paromomycin and miltefosine), for the treatment of resistant cases [5]. A new drug, sitamaquine is currently under development for the potential treatment of visceral leishmaniasis (VL). The use of some of these drugs for the treatment of leishmaniasis are affected by factors such as emergence of drug resistance, especially with the pentavalent antimonials [6–11] and challenges of toxicity, short half-life and high cost of drugs, as well as failure of patient to comply with treatment [5, 12, 13].

Phenolic compounds, which are secondary plant metabolites found in diet, have been reported amongst other natural compounds to have inhibitory effects against protozoan parasites [14, 15]. The potential of phenolic compounds as leishmanicidal agents have been reported in a number of studies [16–19]. They have been reported to mainly function as antioxidants by chelation of metal ions [20] and removal of free radicals [19]. The metal chelation property of phenolic compounds is mainly by the presence of the ortho-dihydroxy (catechol and galloyl groups) and flavan moiety that exists within the compounds [21]. These moieties, the number and orientation of OH groups and the negative charge density present in some of these phenolic compounds are known iron binding elements [22–25]. Studies have also shown that these compounds can induce apoptotic cell death in *Leishmania* via other pathways other than iron chelation [26, 27].

Iron metabolism is an essential pathway that is important for *Leishmania* parasite survival and replication in the phagolysosomes of macrophages [28–30]. Within the parasitophorous vacuole of macrophages, the parasites have the ability to utilize various iron sources such as heme [31], transferrin [32], lactoferrin [33, 34] and hemoglobin [35]. Iron serves as an internal precursor of Fe-S clusters and Fe-dependent enzymes serving as a cofactor of several enzymes like iron superoxide dismutase (FeSOD) and constituent element of ribonucleotide reductase [30, 36], thus supporting essential cellular functions. Therefore, the selective removal of iron by chelation would probably result in reduction in the accessibility of iron to the parasite which would likely impair growth and eventually cause death of parasites. In this study, we investigated the effect of ten phenolic compounds on promastigotes and intracellular amastigotes of *Leishmania donovani* and suggest a mechanism of their action against the parasite.

## Methods

### Compounds

**Fig 1:**
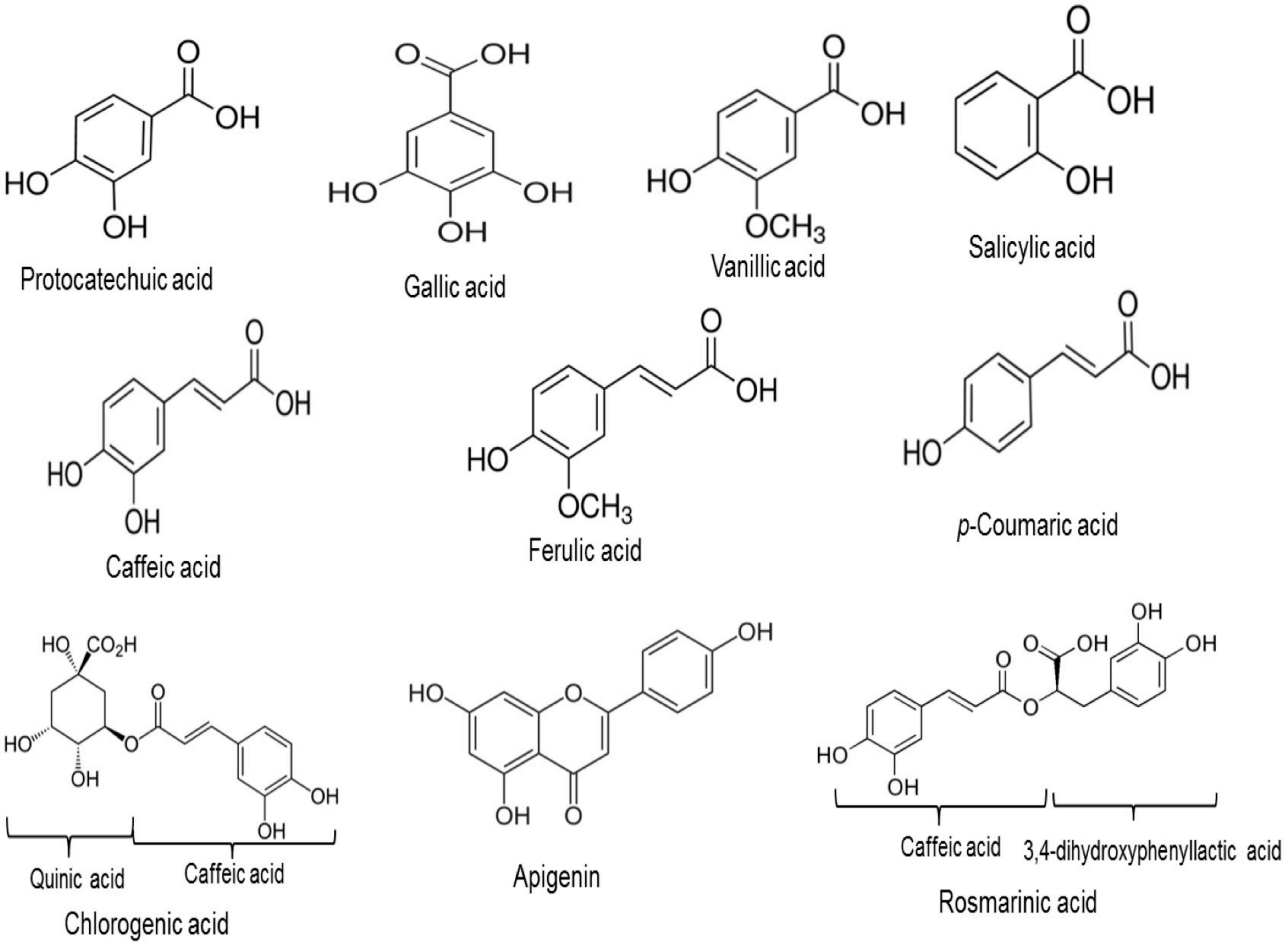
Structures of selected phenolic compounds.

Stock solutions with concentration between 100–730 μM of the phenolic compounds (protocatechuic acid, gallic acid, caffeic acid, vanillic acid, ferulic acid, p-Coumaric acid, apigenin, chlorogenic acid, rosmarinic acid, salicylic acid) (Fig 1) and deferoxamine (Sigma Aldrich, USA) were prepared by dissolving in dimethyl sulfoxide (DMSO) at room temperature and stored at 4°C. The final concentration of DMSO used was 1%. Amphotericin B (Sigma Aldrich, USA) was prepared in double distilled water. Deferoxamine, a known iron chelator and Amphotericin B, a drug used for the treatment of leishmaniasis, were used as controls.

### Parasite and human cells

*Leishmania donovani* promastigotes (MHOM/SD/62/1S strain) were kindly provided by Dr. Yamthe Lauve (Bei Resources NIAID, NIH). The promastigotes were cultured and maintained at 25°C in M-199 medium containing 100 mg/L L-glutamine, 100 μ/ml penicillin-G, 100 μg/mL streptomycin and complemented with 10% heat inactivated fetal bovine serum (FBS). RAW 264.7 (RIKEN BioResource Centre Cell Bank, Japan) cells were kindly provided by Professor Regina Appiah-Opong of the Clinical Pathology Department, Noguchi Memorial Institute for Medical Research, Ghana.

### Promastigote viability assay

The MTT assay was done as previously described [37]. Briefly, promastigotes (2 x 10^5^ cells) were cultured in freshly prepared M-199 medium supplemented with 10% heat inactivated fetal bovine serum in the presence or absence of varying concentrations of the phenolic compounds (0–50 μg/mL) for 72 hours at 25°C. Amphotericin B (0–50 μg/mL) and deferoxamine (0–50 μg/mL) were used as positive controls. After incubation, 5 mg/mL MTT solution was added to the promastigotes in each well and incubated for 3 hours at 25°C. In this assay, the yellow tetrazolium MTT dye was reduced to insoluble formazan crystals (purple color) in living cells using NADH as their reducing agent. Formazan crystals formed after incubation were solubilized with acidified isopropanol and incubated at 37°C for 30 min. The change in color from yellow to purple was read at an absorbance of 570 nm using Thermo Scientific Varioskan LUX. The cell viability and IC_50_ were determined from the concentration response curve generated using GraphPad Prism 6.0 Software.

### Parasite growth analysis

The dose dependent inhibition of phenolic compounds on promastigotes was determined by culturing promastigotes (2 x 10^5^ cells) in freshly prepared M199 medium in the presence and absence of varying concentration of the test compounds, apigenin (IC_50,_ 2x IC_50,_ 4x IC_50_), rosmarinic acid (IC_50,_ 2x IC_50,_ 4x IC_50_), amphotericin B (1/2 IC_50,_ IC_50_) and deferoxamine (IC_50,_ 2x IC_50,_ 4x IC_50_), for 120 hours. The number of parasites was determined by staining with trypan blue every 24 hours. Quantification of the viable parasites was determined by counting the parasites with the clear cytoplasm (non-stained) using a Neubauer hemocytometer with a cover slip. Three independent experiments were performed, and data expressed as mean ± standard deviation.

### Morphology analysis

Promastigotes (2 x 10^6^ cells) were incubated with appropriate concentrations of apigenin (2x IC_50_), rosmarinic acid (2x IC_50_), amphotericin B (1/2 IC_50_) and deferoxamine (2x IC_50_) for 24 hours at 25°C, harvested by low-speed centrifugation at 239 x g for 10 min, washed once with Voorheis Modified Phosphate Buffered Saline (vPBS), fixed in 8% paraformaldehyde (PFA), lysed with 10% v/v Triton X-100 and incubated for 10 min. The cell pellets were then washed twice and resuspended in 1x PBS. The cells were then allowed to settle on poly-L-lysine coated slides and then rinsed with 1x PBS. Tris-HCl (100 mM) pH 7.5 in 1x PBS was used to block the PFA for 10 minutes. DAPI stain (0.1μg/mL) was added followed by one wash in 1x PBS. Coverslips were mounted onto the slides using Vectashield mounting media and observed under the Olympus fluorescent microscope to detect any phenotypic changes in *L*. *donovani* promastigotes. The total number of parasites was determined by direct counting of parasites per field, using ImageJ Software. The frequency of parasites was visually determined by counting parasites that had any abnormalities in their structure compared to the untreated control.

### Cell cycle analysis

Cell cycle analysis of treated and untreated *L*. *donovani* promastigotes was performed using flow cytometry. After treatment of promastigotes (2 x 10^6^ cells) with the appropriate concentrations of the compounds [apigenin (2x IC_50_), rosmarinic acid (2x IC_50_), amphotericin B (1/2 IC_50_) and deferoxamine (2x IC_50_)] for 24 hours, the cells were harvested and fixed in 70% ethanol (diluted in 1x PBS) for 1 hour. The fixed cells were harvested, resuspended in 1x PBS for 1 min and washed twice in 1x PBS. Staining solution (Guava cell cycle reagent) was added and incubated at room temperature for 30 min. The percentage cell count in G0, G1, S, G2 and M phases of the cell cycle were determined with BD LSR Fortessa™^.^ X-20 analyzer (BD Biosciences) and data was analyzed with FlowJo software.

### Analysis of promastigote mitochondria integrity

Promastigotes (4 x 10^6^ cells) were treated with apigenin (2x IC_50_), rosmarinic acid (2x IC_50_), amphotericin B (1/2 IC_50_) and deferoxamine (2x IC_50_)] and then incubated for 24 hours at 25°C. After incubation, the promastigotes were harvested and resuspended in serum-free M199/1%BSA. MitoTracker Red CMXROS (a cationic fluorescent dye that labels the mitochondria within live cells) at 100 nM was added and incubated for 30 min. Parasites were washed once with serum-free M199/1% BSA, pelleted by centrifugation at 1500 rpm for 10 min, resuspended in 1x vPBS and 6% paraformaldehyde and incubated at 4°C for 1 hour. The promastigote pellets were washed twice and resuspended in 1xPBS. The parasites were then allowed to settle on poly-L-lysine coated slides and DAPI stain (0.1μg/mL) was added followed by one wash in 1x PBS. Coverslips were mounted onto the slides using Vectashield mounting media and observed under the Olympus fluorescent microscope to detect any abnormalities in the structure of the mitochondria. The total number of parasites was determined by direct counting of parasites per field, using ImageJ Software. Parasites with distorted mitochondria were visually determined by counting parasites that had any abnormalities in the structure of the mitochondria compared to the untreated control.

### Iron content analysis

Intracellular iron content of *Leishmania donovani* was estimated by treating promastigotes (2 x 10^7^ cells) with rosmarinic acid (4x IC_50_) and apigenin (4x IC_50_), and amphotericin B (IC_50_), deferoxamine (4x IC_50_) and incubated at 25°C for 24 hours. The parasites were then washed twice with 1x PBS and resuspended in 50 μl deionized water. An aliquot (10 μl) of the parasite suspension was used for protein content determination as described by [38]. The remaining parasite sample (40 μl) was digested with HNO_3_ for 1 hour at 80°C and overnight at 20°C. Cell digestions were stopped by the addition of 30% H_2_O_2_ and topped up to 1.5 ml with deionized water. Iron content in digested samples, spent and unused media were measured by atomic absorption spectroscopy using a Perkin Elmer Analyst 300 spectrometer.

### RNA extraction and quantitative real time polymerase chain reaction

Promastigotes (1 x 10^7^ cells) were separately treated with rosmarinic acid, apigenin, amphotericin B and deferoxamine, harvested after 24 hours incubation at 25°C. The parasites were then pelleted by centrifugation at 239 × g for 10 min and the pellet was re-suspended in 1 mL TRIzol. Samples were incubated overnight at -80°C and total RNA was extracted as described by the manufacturer’s protocol using Quick-RNA MiniPrep Plus (Zymo Research, USA). Purity of total RNA was determined to be approximately 2.0 using a Nanodrop. The oligonucleotide primers for the amplification of the iron dependent enzymes—ribonucleotide reductase (RNR) (3’TACGACACGCTGAAGGAGTG and 5’CAACAACTTCTGCGCATCG), iron superoxide dismutase A (FeSOD) (3’GCTCGGCTTCAACTACAAGG and 5’GTCCGTGAAGGTCTTCTTCC) and *Leishmania* iron transporter 1 (LIT1) (3’CCTACTCACTTGGCCTGCAT and 5’ TAGCAGCTGTGTCTGTCGTC) were designed using sequences of *Leishmania* reference genome from tritrypdb.org (http://tritrypdb.org/tritrypdb/) and Primer 3 software. The oligonucleotide primers used in amplifying the housekeeping gene Histone 2A were (3’ CGCGAAATGTGGTCTGATCT and 5’TCTTCGCTCGACTACAGCAG). The RT-qPCR reaction was conducted as described by manufacturer’s protocol with final concentrations of oligonucleotide primers as 0.4 μM, 1x Luna Universal One-Step Reaction mix, 1x Luna WarmStart RT Enzyme Mix and template RNA ≤ 1 μg. All the RT-PCR products were standardized with respect to the endogenous control, Histone 2A. The levels of the mRNA transcripts were obtained using the QuantStudio 5 Real-Time PCR System (Applied Biosystems). The data represent three independent experiments done in triplicate and a fold change in gene expression of >2 was considered significant.

### Determination of cytotoxicity of compounds on mammalian cell

Raw 264.7 cells were seeded into 96-well plates at (1 x 10^6^ cells) in DMEM supplemented with 10% FBS, 100 μg/mL streptomycin, 100 u/mL penicillin-G, 2g/L NaHCO_3_ and incubated at 37°C for 24 hours in 5% CO_2_. The cells were treated with 0–50 μg/mL concentrations of the phenolic compounds for 48 hours at 37°C in 5% CO_2_. Deferoxamine and amphotericin B at 0–50 μg/mL concentrations were used as positive controls. Cell viability was estimated by measuring the mitochondria oxidative activity by MTT assay and absorbance was read at 570 nm using Thermo Scientific Varioskan LUX. Selectivity index (SI) was calculated as the ratio of the CC_50_ to IC_50_ values.

### Analysis of iron chelation in amastigotes

Amastigote cultures were prepared as reported by Jain and colleagues [39]. Briefly, Raw 264.7 cells (4 x 10^7^ cells) were seeded in 4 chamber slides and 1 x 10^7^ cells in 96 well plates and incubated for 24 hours at 37°C in 5% CO_2_. The RAW 264.7 cells were infected with metacyclic promastigotes (1 x 10^8^ cells) at an infection ratio of 1:10 macrophage: parasites and incubated for 24 hours at 37°C in 5% CO_2._ The overlying medium was removed, and the monolayer intracellular amastigotes were carefully washed four times with serum free M199 to remove free parasites. Freshly prepared M199 containing 10% FBS and the test compounds at concentrations ranging from 0–50 μg/mL were added to the infected cells and incubated for 48 hours at 37°C in 5% CO_2._ Triplicate incubations were performed in all the experiments. The chamber slides were washed thrice after incubation with M199 without FBS. The slides were then fixed in absolute methanol for 30 sec, rehydrated in 1x PBS for 5 min and stained with DAPI for 15 min. The slides were viewed by fluorescence microscopy and the number of infected macrophages counted using ImageJ Software to determine the infectivity index and IC_50_ values for amastigotes.

The **infected RAW 264.7** cells in the 96 wells microtiter plates were also incubated for 48 hours and washed thrice as above. Sodium dodecyl sulfate (SDS) of concentration 0.05% was added to the wells for 30 sec for controlled lysis with M199 and 10% FBS. After the 30 sec, M199 with 10% FBS was added and the plates incubated at 25°C for 72 hours for the differentiation of the rescued amastigotes to promastigotes. Following incubation, the amastigotes were subjected to MTT assay and the absorbance obtained were used to estimate IC_50_ values of the compounds against the amastigote forms.

### Statistical analysis

All results obtained were represented as mean±standard deviation (SD) from three independent experiments. GraphPad Prism 6.0 Software was used in the analysis of the data. The *in vitro* leishmanicidal activity, indicated as IC_50_, was derived from non-linear regression analysis. Statistically significant differences for the different groups were determined by Student’s t test and Dunnett’s multiple comparisons test, p-value ≤ 0.05 were considered significant. Cell cycle data was analysed with FlowJo Version 10 and GraphPad Prism 6.0 Software. The RT-PCR was done in triplicate and statistical analysis was done using the student t test and the Dunnett’s multiple comparisons test, p-value ≤ 0.05 were considered significant.

## Results

### Effect of phenolic compounds on the replication of *Leishmania donovani* promastigotes

To investigate the effects of the phenolic compounds on cell viability, 2 x 10^5^ promastigotes were incubated with varying concentrations (0–50 μg/mL) of the phenolic compounds. The phenolic compounds that elicited significant anti-leishmanial activity were rosmarinic acid and apigenin. Treatment with rosmarinic acid and apigenin affected parasites’ growth in a dose- and time-dependent manner with IC_50_ values of 16.34 ± 0.1 μM and 22.77 ± 0.01 μM respectively. Amphotericin B and deferoxamine had IC_50_ values of 6.56 ± 0.06 μM and 10.35 ± 0.01 μM respectively (Table 1).

**Table 1:** Anti*-*leishmanial effect and selectivity indexes of phenolic compounds on promastigotes.

Compounds	Promastigotes IC_50_ (μM) ± SD	Macrophages CC_50_ (μM) ±SD	Selectivity Index (CC_50_/IC_50_)
**Hydroxycinnamic acid**			
p-Coumaric acid	76.09±0.01	322.60±0.15	4.24
Ferulic acid	69.00±0.01	346.70±0.01	5.02
Caffeic acid	58.77±0.01	129.92±0.01	2.21
Vanillic acid	68.97±0.02	13.15±0.01	0.19
**Hydroxybenzoic acid**			
Gallic acid	198.00±0.03	33.76±0.02	0.17
Protocatechuic acid	72.54±0.01	511.90±0.01	7.05
Salicylic acid	75.40±0.02	62.99±0.01	0.83
**Phenolic acid esters**			
Rosmarinic acid	16.34±0.1	245.59±0.3	15.03
Chlorogenic acid	53.92±0.03	59.90±0.02	1.11
**Flavonoid**			
Apigenin	22.77±0.01	237.90±0.03	10.45
**Others**			
Amphotericin B	6.56±0.06	15.75±0.03	2.27
Deferoxamine	10.35±0.01	26.88±0.02	2.60

**Fig 2:**
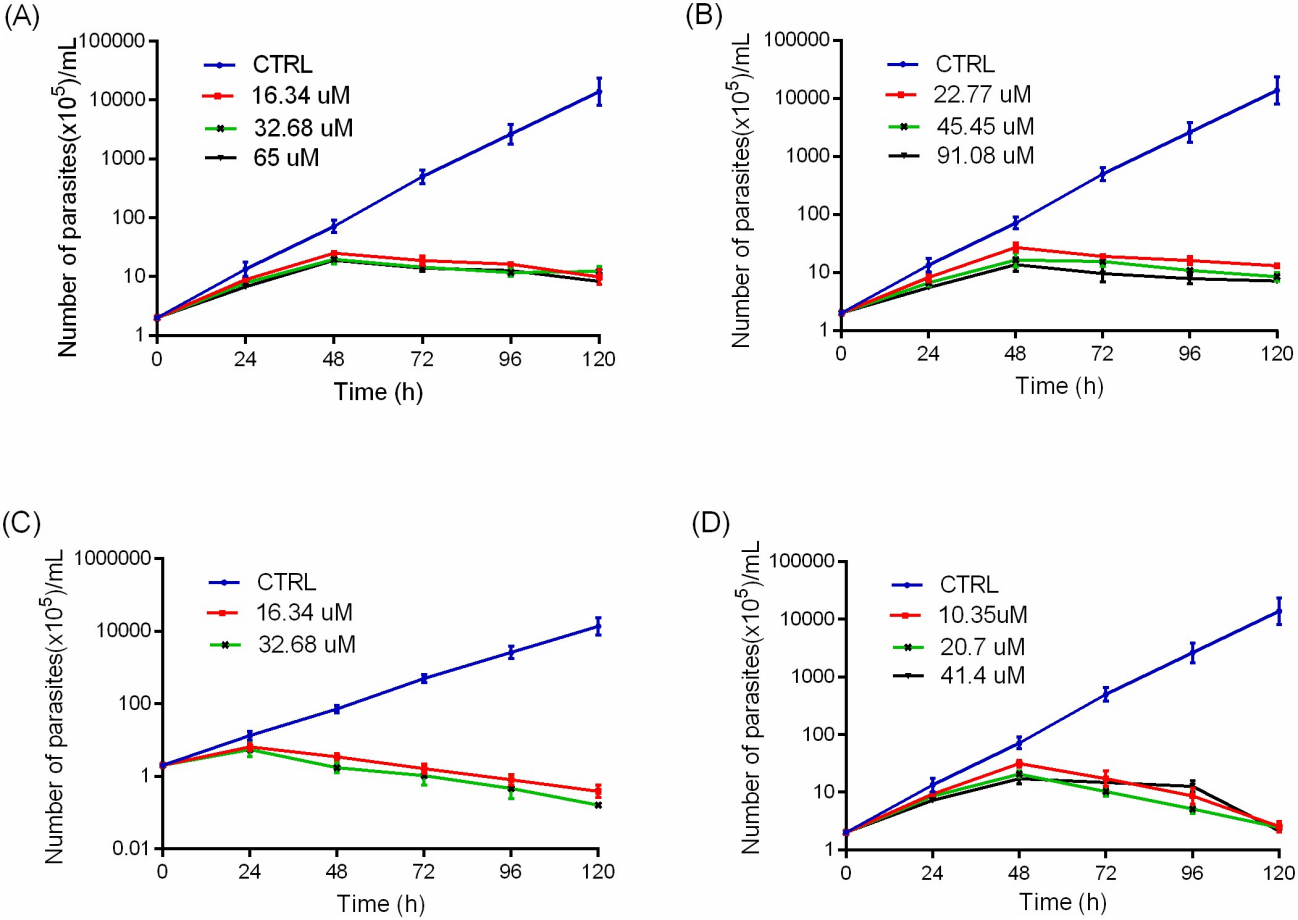
Growth kinetics of *L*. *donovani* promastigotes. Cells were treated with different concentrations of (A) rosmarinic acid (B) apigenin (C) amphotericin B and (D) deferoxamine. The growth kinetics curve was determined by counting parasites every 24 hours over a period of 120 hours. CTRL = control. Points represent mean ± SD from three independent experiments.

In order to evaluate the effects of rosmarinic acid and apigenin on parasite replication, promastigotes were cultured in the presence or absence of the compounds. Cell density was measured every 24 hours over the 120 hours of culturing by staining with trypan blue and counting in a hemocytometer chamber. Treatment with increasing concentrations of apigenin and rosmarinic acid, and the control compounds, amphotericin B and deferoxamine, resulted in drastic reduction in promastigote replication (Fig 2). An exponential growth was observed for untreated cells, while a reduction in cell growth was evident at 48 hours after treatment with rosmarinic acid, apigenin and deferoxamine and at 24h in amphotericin B treated-parasites. At 48 hours there was a 50% decrease in the number of promastigotes when cultured in the presence of IC_50_ concentrations of rosmarinic acid (p value = 0.011), apigenin (p value = 0.024) and deferoxamine (p value = 0.030). The number of parasites reduced significantly by 90% at 24 hours in the presence of IC_50_ concentrations amphotericin B (p value = 0.004) (Fig 2).

### Cytotoxic activity of phenolic compounds on mammalian cells

The cytotoxic effect of compounds on Raw 264.7 cells was assessed using the MTT assay. The phenolic compounds had cytotoxic concentration (CC_50_) values and selectivity indexes (SI) between 13–520 μM and 0.2–15 respectively. Rosmarinic acid and apigenin which had high anti-leishmanial activity gave SI values of 15.03 and 10.45 against the promastigotes, compared with 2.27 and 2.60 SI values of amphotericin B and deferoxamine respectively. Against the intracellular amastigotes, rosmarinic acid, apigenin, deferoxamine and amphotericin B showed SI values of 12.70, 5.21, 0.73 and 2.40 respectively (Tables 1 and 2).

**Table 2:** Anti-leishmanial activity and selectivity indexes of phenolic compounds on amastigotes.

Compounds	Amastigotes IC_50_ (μM) ± SD	Macrophages CC_50_ (μM) ±SD	Selectivity Index (CC_50_/IC_50_)
**Hydroxycinnamic acid**			
p-Coumaric acid	>100	322.60±0.15	ND
Ferulic acid	54.98±0.01	346.70±0.01	6.30
Caffeic acid	56.77±0.01	129.92±0.01	2.29
Vanillic acid	69.97±0.02	13.15±0.01	0.19
**Hydroxybenzoic acid**			
Gallic acid	54.65±0.25	33.76±0.02	0.60
Protocatechuic acid	>100	511.90±0.01	ND
Salicylic acid	90.65±0.04	62.99±0.01	0.69
**Phenolic acid esters**			
Rosmarinic acid	19.21±0.01	245.59±0.3	12.70
Chlorogenic acid	53.75±0.4	59.90±0.02	1.11
**Flavonoid**			
Apigenin	45.66±0.01	237.90±0.03	5.21
**Others**			
Amphotericin B	6.49±0.03	15.75±0.03	2.40
Deferoxamine	36.74±0.01	26.88±0.02	0.73

ND—Not determined

### Effects of compounds on the morphology and mitochondria integrity of *L*. *donovani* promastigotes

**Fig 3:**
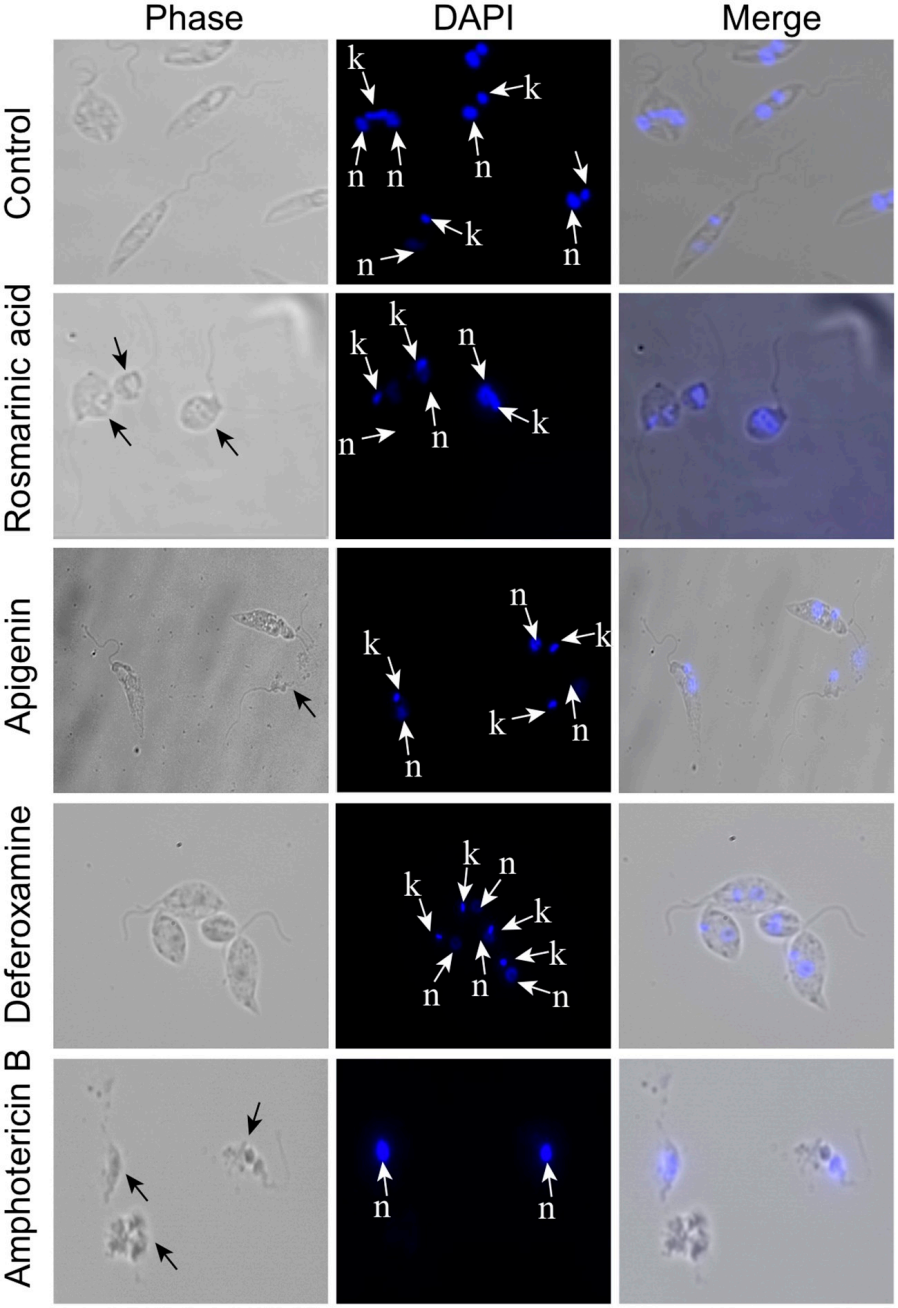
Effect of phenolic compounds on cell morphology. Promastigotes were cultivated in the presence or absence of test compounds (apigenin, rosmarinic acid) and control compounds (amphotericin B, deferoxamine) for 24 hours, stained with DAPI and examined by phase contrast microscopy and fluorescence microscopy at x100. Nucleus—n, Kinetoplast—k.

Morphological changes were assessed after 24 hours in promastigotes treated with 2x IC_50_ values of rosmarinic acid, apigenin and deferoxamine, as well as half the IC_50_ of amphotericin B. Abnormalities in the size and shape of promastigotes relative to the untreated control were observed in the micrographs. About 90% of untreated parasites appeared thin and elongated with long flagellum and smooth cell membrane. The kinetoplast and nuclear DNA also remained intact when observed using the DAPI stain. Upon treatment with rosmarinic acid, 70% (p value = 0.007) of the parasites appeared rounded with associated cytoplasmic condensation and the kinetoplast and nuclear DNA appeared aggregated. Treatment with amphotericin B had 90% (p value = 0.004) of parasites showing irregular shape, severe distortion in the cell membrane with a loss of the kinetoplast DNA, 30% (p value = 0.021) of parasites treated with apigenin appeared ruptured and 90% (p value = 0.005) of promastigotes treated with deferoxamine had rounded morphology with aggregated kinetoplast and nuclear DNA, similar to that observed for rosmarinic acid (Fig 3).

**Fig 4:**
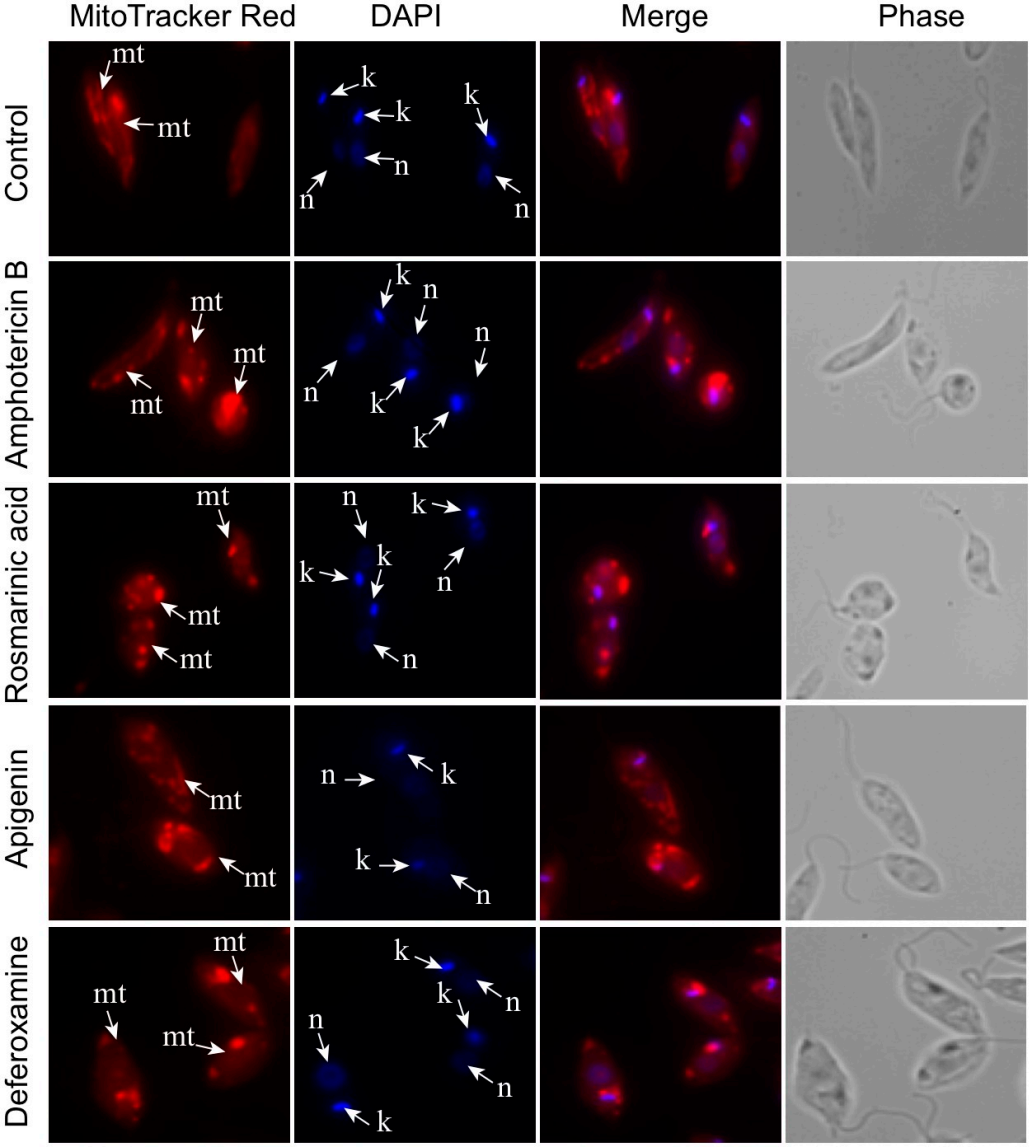
Effects of compounds on the structure of mitochondria. Promastigotes were cultivated in the presence and absence of rosmarinic acid, apigenin, amphotericin B and deferoxamine for 24 hours and stained with MitoTracker Red CMXROS and DAPI, examined by phase contrast and fluorescence microscopy at x100. Mitochondria—mt, Nucleus—n, Kinetoplast—k.

We further investigated the effect of rosmarinic acid and apigenin on the structure of the mitochondria by incubating the promastigotes in the presence or absence of the compounds for 24 hours. The parasites stained with mitotracker red and DAPI revealed that about 98% of the untreated parasites had a well-defined mitochondrion within the cell body of the parasite. When parasites were treated with rosmarinic acid, apigenin, deferoxamine and amphotericin B, 70% (p value = 0.037), 75.8% (p value = 0.039), 81% (p value = 0.004) and 99% (p value = 0.0036) respectively of promastigotes had the mitotracker dye accumulating in the cytoplasm with bright red aggregation (Fig 4). The normal well-defined shape of the mitochondrion was distorted in treated promastigotes.

### Effect of compounds on the cell cycle of *L*. *donovani*

**Fig 5:**
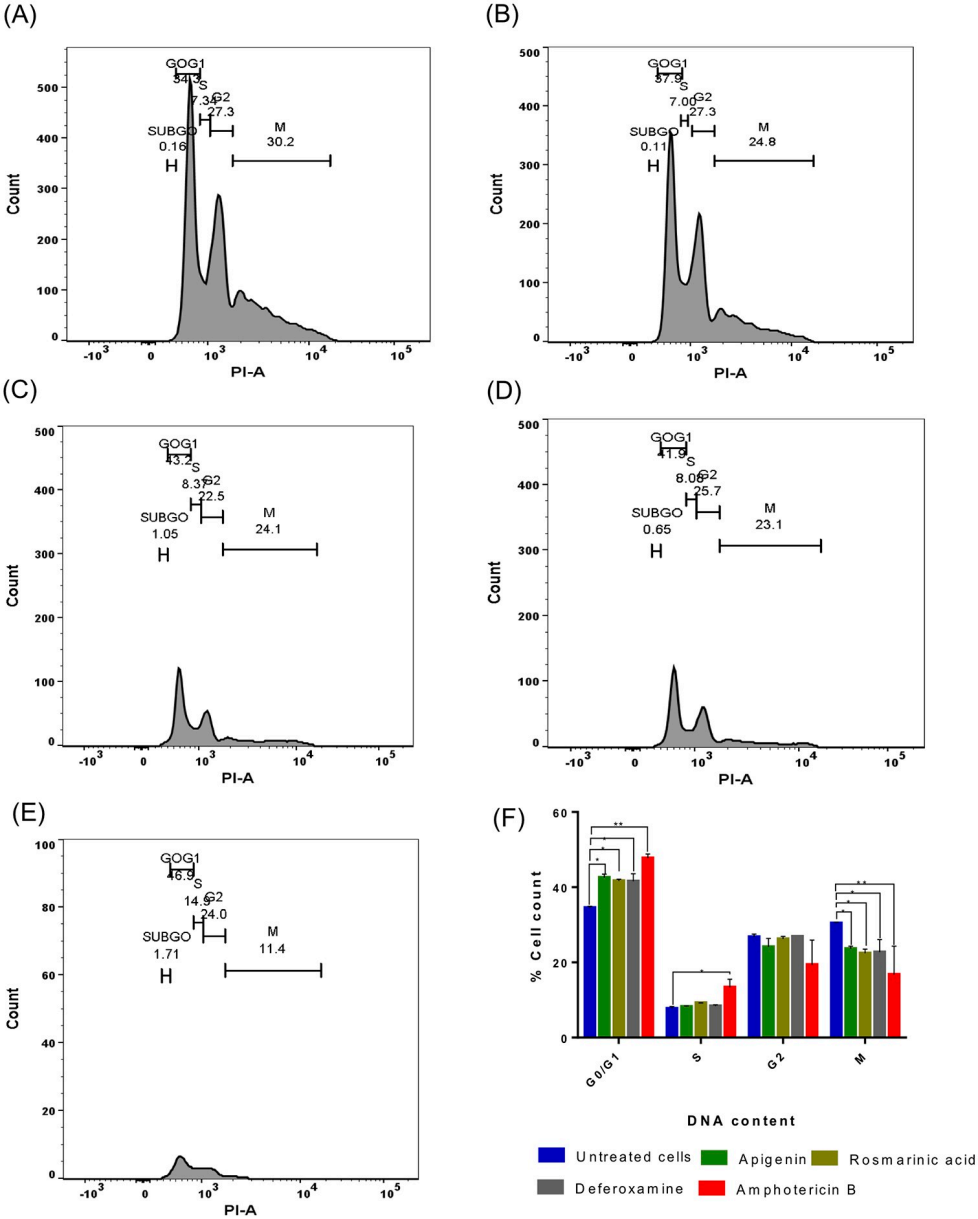
Cell cycle progression of *L*. *donovani*. Flow cytometry histograms of (A) untreated parasites, (B) apigenin, (C) rosmarinic acid, (D) deferoxamine and (E) amphotericin B after 24 hours incubation. Bar charts (F) are the quantification of cell count in G0/G1, S, G2 and M phases of parasites treated with respective concentrations of the compounds. *P<0.05, **P<0.005 were considered significant compared to the untreated promastigotes using Student’s t test.

To investigate the effect of phenolic compounds on the cell cycle of the parasite, the DNA content of treated and untreated cells incubated for 24 hours were analysed using propidium iodide and the cell cycle progression determined by flow cytometry (Fig 5). The untreated parasites had 34.6% of cells at G0/G1 while significant differences were observed in the number of parasites for all the compounds tested. There was an increase in the G0/G1 cells when parasites were treated with apigenin and rosmarinic acid (apigenin 8% increase, p value = 0.008 and rosmarinic acid 7.1% increase, p value = 0.006) (Fig 5B and 5C). We observed a similar increase in deferoxamine and amphotericin B treated parasites (deferoxamine 7% increase, p value = 0.040 and amphotericin B 13.1% increase, p value = 0.0004). Only amphotericin B showed a significant increase in the number of parasites at the S phase. There was no significant change in the number of parasites at the G2 phase for all compounds tested. However, there was a significant decrease in the number of parasites at the M phase for all treatments compared to the untreated (apigenin 6.8% decrease, p value = 0.0062; rosmarinic acid = 8.0% decrease, p value = 0.011; deferoxamine 7.7% decrease, p value = 0.012 and amphotericin B = 13.8%, p value = 0.003) (Fig 5F).

### Effect of apigenin and rosmarinic acid on the intracellular iron content of *L*. *donovani*

**Fig 6:**
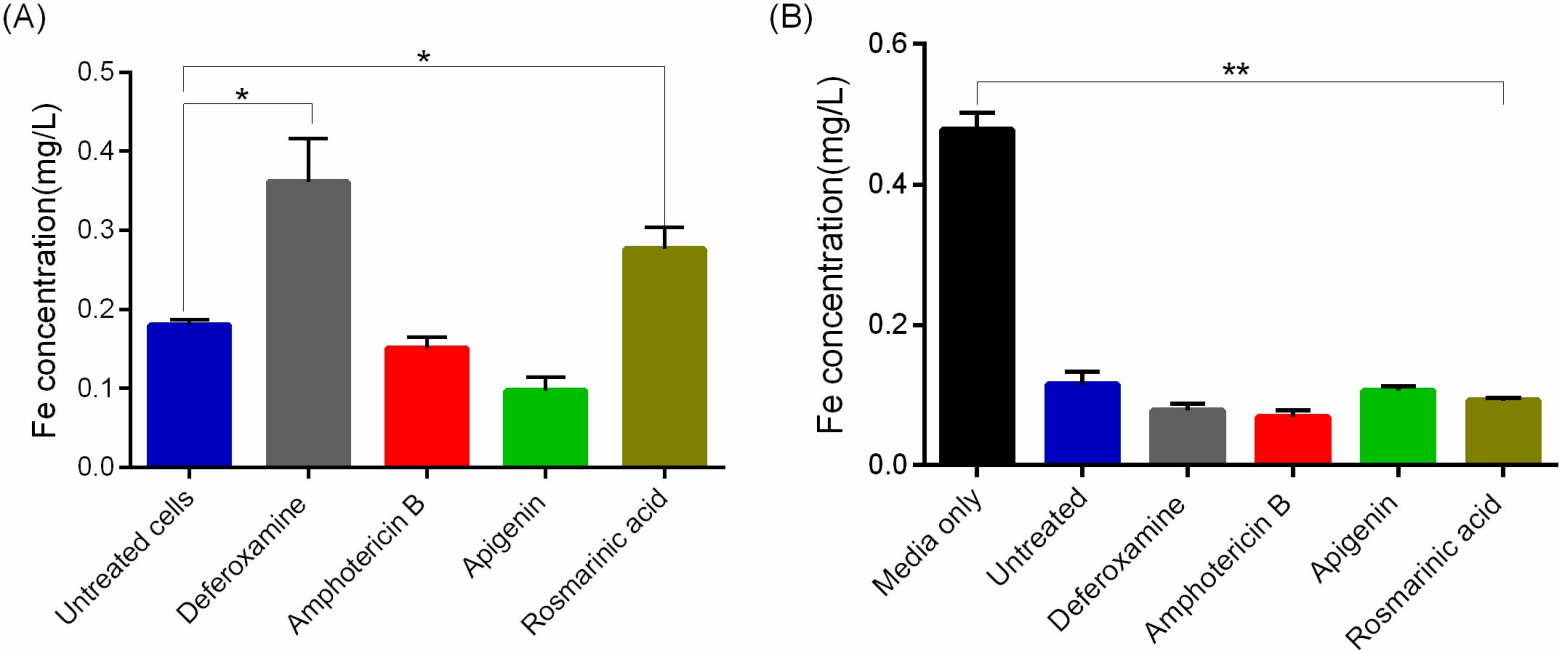
Iron content analyses. (A) Intracellular iron content in promastigotes, (B) iron content in unused and spent culture medium was determined by atomic absorption spectrophotometry. Error bars represents five technical replicates of two independent test. *P <0.05, **P<0.005. Student’s t test and Dunn’s multiple comparison test was used to analyse the data.

In order to assess whether apigenin and rosmarinic acid were acting as iron chelators, the iron concentration in cell digest, unused and spent culture medium was determined with an atomic absorption spectrophotometer as described by Dragset and colleagues [40]. Protein content for the parasites was determined to be 40.5 μg/mL. Promastigotes treated with deferoxamine and rosmarinic acid showed elevated intracellular iron levels relative to the untreated cells (deferoxamine 0.36 mg/L, p value = 0.040 and rosmarinic acid 0.30 mg/L, p value = 0.020). Amphotericin B (0.15 mg/L, p value = 0.120) did not show any significant change in intracellular iron levels, whereas apigenin showed significant decrease 0.1 mg/L, p value = 0.020 compared to the untreated parasites (Fig 6A). However, the concentration of iron in the spent media was significantly decreased by about three-fold in treated parasites compared to the unused (apigenin 0.11 mg/L, p value = 0.002; rosmarinic acid 0.09 mg/L, p value = 0.002; deferoxamine 0.08 mg/L, p value = 0.002; amphotericin B 0.07 mg/L, p value = 0.002) (Fig 6B).

### Effect of rosmarinic acid and apigenin on mRNA levels of iron dependent proteins

**Fig 7:**
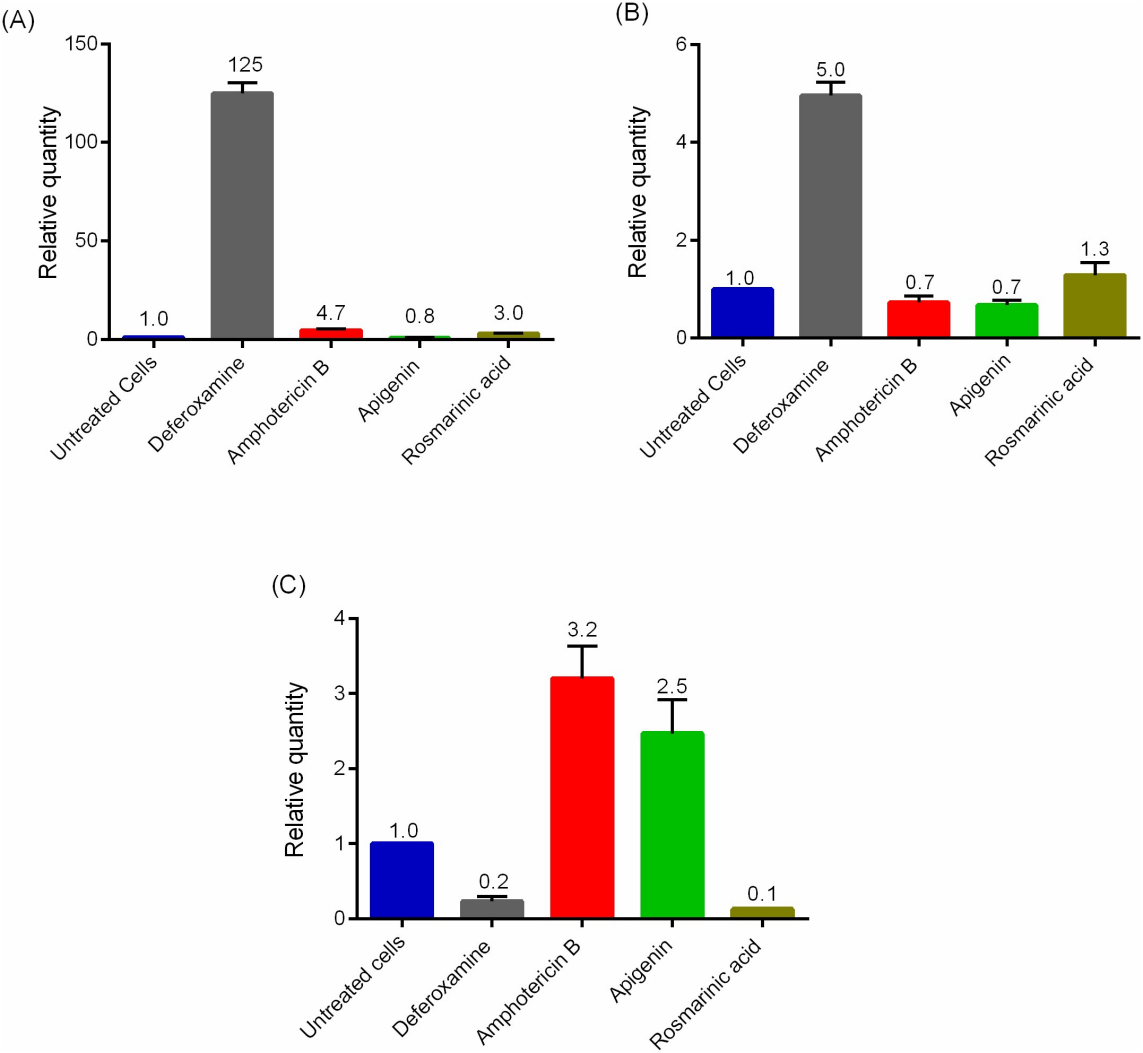
Gene expression of iron dependent proteins. Expression of (A) LIT1, (B) FeSOD and (C) RNR were determined by qRT-PCR. Promastigotes were cultured with the compounds for 24 hours. The data presented is from three independent experiments.

The effect of the test compounds on the mRNA of iron dependent proteins in promastigotes was investigated by determining the mRNA expression levels using quantitative RT PCR. The change in the levels of mRNA expression was determined by comparing with the untreated control. There were varying degrees of expressions of the iron dependent proteins in the presence of the compounds. LIT1 and FeSOD were significantly over expressed in deferoxamine treated parasites compared to the untreated parasites (LIT1 = 125-fold and FeSOD = 5-fold) (Fig 7A and 7B). LIT1 level was also significantly upregulated in amphotericin B (5-fold) and rosmarinic acid (3-fold) treated parasites while there was no observed change in apigenin treated parasites (Fig 7A). The FeSOD levels however did not significantly change in amphotericin B, apigenin and rosmarinic acid treated parasites (Fig 7B). There was an increased expression of RNR in amphotericin B (3-fold) and apigenin (3-fold) treated cells and a decrease in the expression of RNR in deferoxamine (80% reduction, p value < 0.0001) and rosmarinic acid treated parasites (90% reduction, p value < 0.0001) (Fig 7C).

### Effect of phenolic compounds on intracellular amastigotes

**Fig 8:**
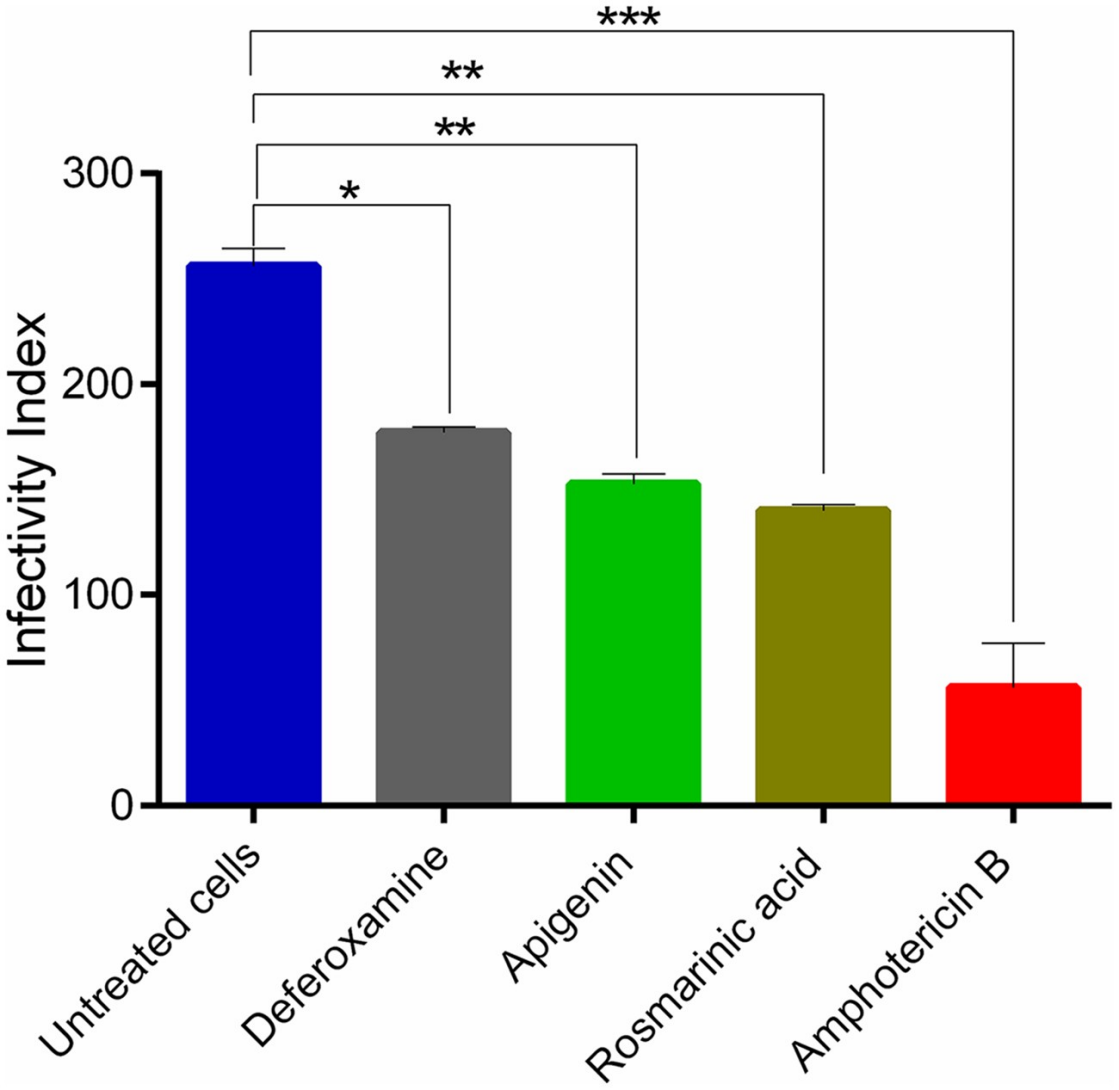
Infectivity index of compounds. The parasite load in infected macrophages was determined by counting the number of intracellular amastigotes per 100 macrophages. The data represents the mean±SD of three independent experiments in triplicate *P<0.05, **P<0.005, ***P≤0.0005 were considered significant, compared to the untreated cells using student’s t test.

To assess the effects of the phenolic compounds on amastigotes, infected macrophages were treated with increasing concentration of the compounds (3.12–50 μg/mL). Rosmarinic acid and apigenin showed significant inhibitory effect against the amastigotes with IC_50_ values of 19.21±0.01 μM and 45.66±0.01 μM respectively. The IC_50_ values observed for amphotericin B and deferoxamine were 6.491±0.03 μM and 36.74±0.01 μM respectively (Table 2). When the infected macrophages were treated with increasing concentrations of rosmarinic acid, apigenin, deferoxamine and amphotericin B, there was a decrease in the number of infected cells. Treatment with amphotericin B at a low concentration (3.12 μg/mL) gave the lowest infectivity index of 56 (p value = 0.0005) compared to the untreated infected macrophages (Fig 8). The infectivity values for rosmarinic acid, apigenin and deferoxamine were lower (rosmarinic acid 140, p value = 0.003; apigenin 152.2, p value = 0.005 and deferoxamine 177, p value = 0.006) compared to the untreated infected macrophages (Fig 8). Surprisingly, there was no visible morphological changes in the amastigotes after drug treatment.

## Discussion

The ability of phenolic compounds known to have effects against certain protozoan parasites [26, 41, 42] was investigated to understand their mechanism of action against *Leishmania donovani*. All the phenolic compounds used were found to be active in the concentration range of 16–198 μM, with rosmarinic acid and apigenin having a high inhibitory effect on both forms of the parasite, with selectivity values higher than amphotericin B, an anti-leishmanial drug and deferoxamine, an iron chelator. The differences in the leishmanicidal activities observed against the parasite between the selected phenolic compounds, and the amphotericin B and deferoxamine control could be explained by the structural variations in these phenolic compounds. Rosmarinic acid has been shown to inhibit the cellular replication of *L*. *amazonensis*, an activity that has been reported to be due to the iron chelating ability of the catechol groups it contains [18]. Caffeic acid, a catechol compound and a component of rosmarinic acid, had a lower inhibitory effect against the parasite compared to rosmarinic acid. The conjugation of caffeic acid with 3-(3,4-dihydroxyphenyl) lactic acid resulting in two iron chelating catechol groups as found in rosmarinic acid could account for its ability to better scavenge iron from the parasite and hence the more pronounced growth inhibitory effect. Apigenin has no catechol functionality in its structure but it has been suggested that the 2–3 double bond and three hydroxyl groups of apigenin might be involved in the generation of reactive oxygen species which inhibited the growth of *L*. *amazonensis* [27, 43, 44]. These groups have also been shown to influence the metal scavenging activity of apigenin [45]. Deferoxamine had a higher inhibitory effect on the parasite compared to the phenolic compounds and has also been shown to have a higher affinity for iron [46, 47]. Its mechanism of scavenging iron from the parasite could result in the inhibitory effect observed.

Rosmarinic acid and apigenin showed significant effects on the morphology of the parasite. The rounding and cytoplasmic condensation of cells treated with rosmarinic acid and the associated aggregation of the kinetoplast and nuclear DNA, could be the way the compound exert its inhibitory effects. Similar morphological changes were observed for deferoxamine treated cells. The observations suggest that rosmarinic acid and deferoxamine probably chelate the free iron within the medium, creating an iron poor environment that affects the stability of the iron acquisition mRNA transcripts leading to their transformation into axenic amastigotes [48], which appear rounded. Other studies have also shown that, certain conditions such as pH, temperature and iron deficient culture media mimic the intracellular environment of the host cell and cause the transformation of thin elongated promastigotes to rounded forms of axenic amastigotes [49, 50]. The morphological changes induced by deferoxamine and rosmarinic acid could cause programmed cell death as suggested by Islamuddin and others [51]. Cells treated with apigenin appeared lysed with the loss of kinetoplast and nuclear DNA. The lytic action observed could be attributed to apigenin’s ability to lyse certain cell organelles, such as the mitochondria and Golgi apparatus [43]. The distortion of the parasite’s cell membrane by treatment with amphotericin B could be due to its binding to ergosterol in the parasite membrane. This could lead to the formation of pores within the cell membrane, which alter the ion balance, causing cell lysis and death of the parasite [52].

Mitochondria integrity which defines the functionality of the cell was also compromised in the presence of the compounds. The effect of apigenin and rosmarinic acid on the mitochondria membrane potential supports findings of several studies that reported their involvement in inducing apoptotic death of cells through the production of ROS. These studies have shown that alteration in the morphology of the mitochondria can lead to the loss of cell viability [43, 53, 54]. Accumulation of amphotericin B within the parasite has also been shown to auto-oxidize and generate ROS within *L*. *donovani* [55, 56] this could cause a loss in the integrity of the mitochondria membrane. The effect of deferoxamine on the parasites supports a study which reported that iron chelating compounds result in the depletion of iron and affect the ability of FeSODA located in the parasite’s mitochondria to mop up the reactive oxygen species [48]. This removal of iron, an essential cofactor, from FeSODA affects the activity and structure of the mitochondria [57]. The parasites that survived treatment with higher concentration of compounds could either be resistant parasites or parasites that have some dysfunction in their mitochondria but are still viable.

The accumulation of cells in GO/GI phase observed when promastigotes were treated with rosmarinic acid, apigenin and deferoxamine suggests that the compounds could be inducing cell cycle arrest by affecting certain cell cycle regulatory proteins. Iron chelation has been reported to decrease the level of expression of cyclin and cdk genes and an increase in cyclin dependent inhibitors [58]. The accumulation of cells in the GO/G1 phase observed for rosmarinic acid treated parasites maybe as a result of inhibition of the replication of cells, halt DNA synthesis and the activity of some cell cycle proteins [59]. It is most likely that, apigenin alters the cell cycle regulatory system by decreasing the expression of cyclins and cdks [60] hence the accumulation of cells in the G0/G1 phase. Microarray analysis of deferoxamine treated HL-60 leukaemia cells resulted in a significant decrease in the mRNA expressions of cyclin genes and RNR [61, 62]. Amphotericin B has been shown to cause an increase in the G1 and S phases, and a decrease in the M phases in cells [63]. The susceptibility of the parasites to amphotericin B has been attributed to the synthesis of sterols initiated in the G1 and S phases of the parasite’s cell cycle [64].

In this study, we observed elevated intracellular iron content of parasites treated with rosmarinic acid and deferoxamine. The presence of pyrozolopyrimidinine (PZP), an iron chelator, caused the accumulation of iron within *Mycobacteria smegmatis*; an obligate intracellular organism like *Leishmania*, indicating that iron chelating compounds could induce an accumulation of iron within cells [40]. Iron depletion from the culture medium and the parasite’s ability to sense iron deprivation could lead to the upregulation of LIT1 and FeSOD and downregulation of RNR for survival and virulence [30]. Rosmarinic acid and deferoxamine might have created an iron depleted environment in the parasite which could have led to the differential expression of these iron acquisition genes in the *Leishmania*. Trypanosomes have been shown to upregulate the transferrin receptor to cope in an iron depleted environment [65]. We observed a 3-fold increase in the expression of *Leishmania* iron transporter 1 (LIT1) in parasites treated with rosmarinic acid compared to the earlier reported six-fold upregulation of the gene in an iron depleted environment [48]. The upregulation of RNR observed for amphotericin B and apigenin treated cells suggest that these two compounds do not prevent the incorporation of cellular iron into newly synthesized iron dependent proteins.

Amongst the phenolic compounds investigated in this study, rosmarinic acid and apigenin had selectivity indexes greater than 10 suggesting that their effects were on the parasite and not on the host cells [66, 67]. This supports other studies reporting the low toxicity levels of rosmarinic acid and apigenin against host cells [18, 44]. It was also observed that amongst the phenolic compounds, rosmarinic acid and apigenin that had pronounced inhibition against the promastigotes were also potent against the intracellular amastigotes. Their effectiveness was shown by the low level of infectivity compared to the untreated infected-macrophages and their IC_50_ values. This suggests that, the compounds might transverse the host cell but are degraded rapidly [68]. Infected cells treated with deferoxamine, had some cells still infected with promastigotes even at the highest concentration. This limitation of deferoxamine could be attributed to its lipophobic nature causing it not to be able to transverse the cell membrane of the host cell to elicit its effect [69]. It was also observed that, the IC_50_ values of rosmarinic acid and apigenin against the promastigotes were lower compared to the intracellular amastigotes, suggesting that, the compounds have relatively easier access to the promastigotes growing freely in the culture medium but, the presence of the host cell reduces their interaction with the intracellular parasite [18].

The present study showed an anti-leishmanial activity of rosmarinic acid and apigenin against *L*. *donovani* promastigotes and intracellular amastigotes and resulted in changes in the mitochondria integrity and morphology of the cells. Rosmarinic acid and apigenin induced cell cycle arrest in the G0/G1 phase. Elevated intracellular iron levels observed in promastigotes treated with rosmarinic acid correlated with differential expression levels of iron dependent genes which could have led to a decrease in the activity of crucial iron-regulated enzymes. The findings suggest that rosmarinic acid could be exerting its inhibitory effect against the parasite via iron chelation which results in changes in cell morphology and the arrest of the cell cycle while apigenin may exert its inhibitory effects by other mechanisms. These findings present useful chemotherapeutic potential of phenolic compounds in Leishmaniasis. Further studies that include the use of a combination of the most active phenolic compounds and the currently used anti-leishmanial drugs will provide information on any synergistic effects of the compounds. Also, further in vivo studies are essential to evaluate the leishmanicidal activity of rosmarinic acid and apigenin against *Leishmania*.
